# Leukocyte invasion of the brain after peripheral trauma in zebrafish (*Danio rerio*)

**DOI:** 10.1038/s12276-022-00801-4

**Published:** 2022-07-13

**Authors:** Xiang-Ke Chen, Joseph Shiu-Kwong Kwan, Gordon Tin-Chun Wong, Zhen-Ni Yi, Alvin Chun-Hang Ma, Raymond Chuen-Chung Chang

**Affiliations:** 1 Laboratory of Neurodegenerative Diseases, School of Biomedical Sciences, LKS Faculty of Medicine, The University of Hong Kong, Pokfulam, Hong Kong SAR, China; 2 Department of Brain Sciences, Imperial College London, London, UK; 3 Department of Anaesthesiology, School of Clinical Medicine, LKS Faculty of Medicine, The University of Hong Kong, Pokfulam, Hong Kong SAR, China; 4 Department of Health Technology and Informatics, Hong Kong Polytechnic University, Hung Hom, Kowloon, Hong Kong SAR, China; 5 State Key Laboratory of Brain and Cognitive Sciences, The University of Hong Kong, Pokfulam, Hong Kong SAR, China

**Keywords:** Neuroimmunology, Acute inflammation

## Abstract

Despite well-known systemic immune reactions in peripheral trauma, little is known about their roles in posttraumatic neurological disorders, such as anxiety, sickness, and cognitive impairment. Leukocyte invasion of the brain, a common denominator of systemic inflammation, is involved in neurological disorders that occur in peripheral inflammatory diseases, whereas the influences of peripheral leukocytes on the brain after peripheral trauma remain largely unclear. In this study, we found that leukocytes, largely macrophages, transiently invaded the brain of zebrafish larvae after peripheral trauma through vasculature-independent migration, which was a part of the systemic inflammation and was mediated by *interleukin-1b* (*il1b*). Notably, myeloid cells in the brain that consist of microglia and invading macrophages were implicated in posttraumatic anxiety-like behaviors, such as hyperactivity (restlessness) and thigmotaxis (avoidance), while a reduction in systemic inflammation or myeloid cells can rescue these behaviors. In addition, invading leukocytes together with microglia were found to be responsible for the clearance of apoptotic cells in the brain; however, they also removed the nonapoptotic cells, which suggested that phagocytes have dual roles in the brain after peripheral trauma. More importantly, a category of conserved proteins between zebrafish and humans or rodents that has been featured in systemic inflammation and neurological disorders was determined in the zebrafish brain after peripheral trauma, which supported that zebrafish is a translational model of posttraumatic neurological disorders. These findings depicted leukocyte invasion of the brain during systemic inflammation after peripheral trauma and its influences on the brain through *il1b*-dependent mechanisms.

## Neurological disorders: White cells invade the brain after peripheral trauma

Invasion of the brain by white blood cells followed tail amputation in zebrafish, the resulting systemic inflammation producing anxiety-like behaviors. Scientists have long recognised an association between systemic inflammation following peripheral traumatic injury such as limb loss and post-traumatic neurological disorders such as anxiety and depression. Raymond Chuen-Chung Chang at the University of Hong Kong, Alvin Chun-Hang Ma at Hong Kong Polytechnic University, China, and co-workers found that following trauma, white cells, mainly macrophages, flowed from neighboring tissues into the hindbrain, before spreading throughout the brain. This influx of white cells, mediated by the small signaling protein interleukin-1b, triggered anxiety-like behaviors such as hyperactivity and avoidance in the zebrafish. The researchers emphasize that the links between systemic inflammation following peripheral trauma and neurological responses require extensive further research.

## Introduction

Whereas the central nervous system (CNS) is considered immune-privileged from the periphery, the roles of peripheral immune cells remain vital in both homeostasis and disease pathogenesis of the CNS^1^. Apart from the immune cells in the meninges, circulatory leukocytes, which are normally absent from the CNS due to the presence of the blood–brain barrier (BBB), are able to invade the CNS parenchyma under both specific physiological states^2,3^ and diseases, such as traumatic injury, stroke, tumor, infection, and neurodegeneration^1,4,5^. Notably, invasion of peripherally derived leukocytes can be either beneficial or detrimental to the CNS and, together with resident immune cells, therefore serves as a promising therapeutic target of these neurological diseases, although peripheral leukocyte invasion is an indirect pathogenesis to most CNS diseases^6,7^. More importantly, it has been reported that leukocyte invasion accounts for the non-CNS-originated neurological disorders (e.g., sickness, anxiety, and cognitive impairments) that occur in peripheral inflammatory diseases, such as infection, obesity, postoperative cognitive dysfunction (POCD), and hepatitis^8–11^. Unlike CNS diseases, leukocyte invasion may be the primary cause of the neurological disorders observed in peripheral inflammatory diseases, which warrants further investigation.

Trauma or traumatic injury is one of the leading causes of death globally, and approximately 70-90% of adults have experienced at least one type of traumatic injury in their life^12,13^. Immune and inflammatory reactions are the main feature of traumatic injury, while failure to resolve severe inflammation can lead to chronic inflammation or systemic inflammatory response syndrome (SIRS), which affects various organs in the body^14^. Neurological disorders have long been recognized in traumatic injuries, such as posttraumatic cognitive impairment, posttraumatic stress disorder (PTSD), POCD, and postoperative delirium (POD)^15,16^. Of the traumatic injuries, traumatic brain injury (TBI) commonly shows adverse cognitive effects along with peripheral leukocyte invasion^16^. However, little is known about leukocyte invasion in non-CNS-originated neurological disorders after peripheral trauma, such as limb injury, which is much more prevalent in the general population, while a pioneering animal study observed brain leukocyte invasion during POCD after peripheral surgical trauma with anesthesia^11^. Although neurological disorders in common peripheral trauma may be milder than TBI and POCD, their relatively high morbidity can also lead to subtle alterations to the CNS. More importantly, the experience of these peripheral traumas can increase the risk of dementia^15^. Thus, this generated interest in exploring the role of leukocyte invasion as a potential etiology of neurological disorders observed in nonsurgical peripheral trauma.

Systemic inflammation or SIRS is initiated shortly after severe traumatic injury, and to a larger extent can be found in nonsurgical trauma, wherein peripheral leukocytes are recruited by cytokines or chemokines and act protectively in the inflamed tissues^17,18^. Nevertheless, activated leukocytes also serve as the source of proinflammatory cytokines in chronic systemic inflammation^18^. This finding suggests the double-edged sword role of invading leukocytes in inflamed tissue during systemic inflammation. Intriguingly, the CNS, with its resident immune cells, is susceptible to being inflamed by peripheral inflammation, which is also known as secondary neuroinflammation^19,20^. This inflammation can be attributed to the crosstalk between the CNS and peripheral immunity through circulating inflammatory mediators, such as cytokines, chemokines, and leukocytes, but it varies between diseases. Many studies have revealed that systemic inflammation-induced neurological disorders are mediated by proinflammatory cytokines, such as interleukin-1β (IL-1β), through multiple dissociable mechanisms, such as neuronal dysfunction and neuronal death^21,22^. Notably, the cytokine-induced brain damage in systemic inflammation can result in both transient and long-term cognitive impairment. However, unlike cytokines, the recruitment and functions of invading leukocytes in secondary neuroinflammation and subsequent neurological disorders in peripheral inflammatory diseases, especially in peripheral trauma, are not well understood and may involve a cytokine-mediated pathogenesis.

Zebrafish (*Danio rerio*), a small tropical fish with great potential in genetic tractability, in vivo cell tracking, and human disease modeling, has emerged rapidly in the past decade as an alternative but critical translational model of leukocyte trafficking in peripheral trauma^23^ as well as various neurological disorders^24^. Here, we exploited live-cell tracking and genetic manipulation in transgenic zebrafish larvae to investigate whether leukocytes invade the brain during systemic inflammation after peripheral trauma and its underlying mechanisms and impacts on the CNS.

## Materials and methods

### Zebrafish strains and maintenance

Wild-type (WT) and transgenic (Tg) zebrafish lines were maintained in a 14:10 h light:dark cycle and were fed with live brine shrimp twice a day. The *Tg(mpeg1:dendra2)* zebrafish line was generated by using the Tol2-mpeg1-dendra2 plasmid (Addgene, 51462) with in vitro transcribed Tol2 transposase mRNA (Addgene, 31831). Embryos from WT, *Tg(coro1a:DsRed)*^23^, *Tg(mpeg1:GFP)*, *Tg(pu.1:EGFP)*, *Tg(mpeg1:dendra2)*, *Tg(kdrl:GFP;coro1a:DsRed)*, and *Tg(kdrl:GFP;mpx:mCherry)* were raised at 28.5 °C and staged as previously described^25^. All animal experiments were conducted in accordance with protocols approved by the Committee on the Use of Laboratory and Research Animals (CULATR) of the University of Hong Kong (4635-17) and the Animal Subjects Ethics Sub-Committee (ASESC) of The Hong Kong Polytechnic University (19-20/46-HTI-R-GRF).

### Peripheral trauma

WT or transgenic zebrafish embryos at 3 days post-fertilization (dpf) were anesthetized using 0.16 mg/ml tricaine (Sigma–Aldrich, A5040), and then the tail amputation assay was applied at a position posterior to the blood circulation of the caudal fin to model peripheral trauma as previously described^26^. After tail amputation, zebrafish embryos were immediately transferred back to sterilized E3 medium (5 mM NaCl, 0.17 mM KCl, 0.33 mM CaCl, and 0.33 mM MgSO_4_, pH 7.4) containing methylene blue.

### Light-sheet imaging

Zebrafish embryos at 3–5 dpf were anesthetized using 0.16 mg/ml tricaine and then mounted in 1.5% low-melting agarose (Sigma–Aldrich, A9045) using a glass capillary for fluorescence imaging by using a Zeiss Lightsheet Z.1 Selective Plane Illumination Microscope with or without a time-lapse mode as previously described^27^.

### Flow cytometry

The head region of WT or *Tg(mepg1:GFP)* zebrafish embryos was isolated and then homogenized and filtered to obtain the cell suspension. A Beckman Coulter FC 500 was utilized to quantify the number and percentage of GFP + cells under various conditions.

### Dendra2 photoconversion

*Tg(mpeg1:dendra2)* zebrafish embryos at 2.5 dpf were anesthetized using 0.16 mg/ml tricaine and then mounted in a confocal dish using 1% low-melting agarose. A Leica SPE confocal microscope was applied to induce photoconversion in the tail region, largely the caudal hematopoietic tissue (CHT), of embryos by using prolonged (approximately 1 min) illumination with a scanned 405-nm laser. For confirmation, the GFP and RFP signals were assessed in both the tail and head regions of the embryos using a Leica SPE confocal microscope after photoconversion.

### Behavioral assay

Zebrafish larvae at 4 days post-trauma (dpt) and their siblings were transferred to a 35-mm Petri dish containing 2 mL of E3 medium. The protocol was adapted from the locomotion assay according to a previous paper^28^. The locomotor activity of zebrafish larvae in different groups were simultaneously recorded for a 20-min period using a camera. A 10-min period after acclimation was analyzed using ImageJ (NIH) and LoliTrack V (Loligo system). The total travel distance, active swim time, mean velocity, and thigmotaxis were calculated as described previously^29^.

### *Spi-1 proto-oncogene b* knockdown

Morpholinos (MOs) were purchased from Gene Tools and microinjected into zebrafish embryos at the one-cell stage: *spi.1b* (*pu.1*)-MO, 0.5 mM (5ʹ-GATATACTGATACTCCATTGGTGGT-3ʹ)^30^, while standard control MO was injected as the control (CTRL). In addition, the *Tg(pu.1:EGFP)* zebrafish line was used to confirm the transient knockdown of *pu.1*.

### Interleukin 1-beta knockout

*il1b* mutant zebrafish were generated via transcription activator-like effector nucleases (TALEN), resulting in a loss of function as previously described^31^. All *il1b* mutations were confirmed by restriction fragment length polymorphism (RFLP) assays using *il1b* genotyping primers (Supplementary Table 1) and NsiI-HF® restriction enzymes (NEB, R3127L), as well as Sanger sequencing.

### Quantitative PCR

Total RNA was extracted from the tail region or the head region of zebrafish embryos using RNAiso Plus (Takara, 9108). cDNA was then synthesized using a RevertAid First Strand cDNA Synthesis Kit (Thermo Fisher, K1621) in accordance with the manufacturer’s protocol. Quantitative PCR (qPCR) was finally performed on an ABI 7300 Real-Time PCR System with FastStart Universal SYBR Green Master reagents (Roche, 04913914001) and corresponding primers (Supplementary Table 1).

### Whole-mount in situ hybridization

Zebrafish embryos were subjected to whole-mount in situ hybridization (WISH) using standard protocols as described previously^32^. DIG-labeled antisense probes were made from the pGEM®-T Easy vector (Promega, A1360) containing the gene-coding sequences via in vitro transcription using a DIG RNA Labeling Kit (Roche, 11175025910). In addition, *il1b* (852 bp) and *il6* (449 bp) antisense probes were designed and synthesized in the present study (Supplementary Table 1).

### Phospho-histone H3 and TUNEL staining

Immunofluorescence was applied to detect the cells undergoing mitosis using phospho-histone H3 (PH3) primary antibody (Cell Signaling Technology, 9701). In addition, an ApopTag® Fluorescein In Situ Apoptosis Detection Kit (Millipore, S7110) was used to detect apoptotic cells in fixed zebrafish larvae following the protocols described previously^30^.

### Acridine orange, neutral red, and LysoTracker staining

Acridine orange (AO) (Sigma–Aldrich, A6014) at 10 µg/ml, neutral red at 2.5 µg/ml, and LysoTracker Red DND-99 (Invitrogen, L7528) at 10 μM in E3 medium were applied to stain 3–4-dpf WT or transgenic live zebrafish embryos.

### Mass spectrometry-based proteomics

Total protein was extracted using cell lysis buffer (Sigma–Aldrich, C3228) from the head region of zebrafish embryos. After purification and trypsin (Promega, V5111) treatment, the peptides were desalted using Pierce C18 Spin Columns (Thermo Fisher, 89870). Proteomics was then performed on a Thermo Fisher Orbitrap Fusion Lumos Mass Spectrometer coupled with a Dionex UltiMate 3000 RSLCnano. Label-free relative quantification was processed with Progenesis QI software, and the abundance of various proteins was quantified based on three independent experiments and normalized according to the housekeeping protein *glyceraldehyde 3-phosphate dehydrogenase* (*gapdh*).

### Statistics

Data are presented as the mean ± standard deviation (S.D.). Independent *t* test, one-way ANOVA, and two-way ANOVA with Tukey’s HSD post hoc test were performed where appropriate using Statistical Package for the Social Sciences (SPSS) Version 14.0, and a *p* value less than 0.05 was regarded as significant.

## Results

### Leukocytes invade the brain after peripheral trauma in zebrafish larvae

**Fig. 1:**
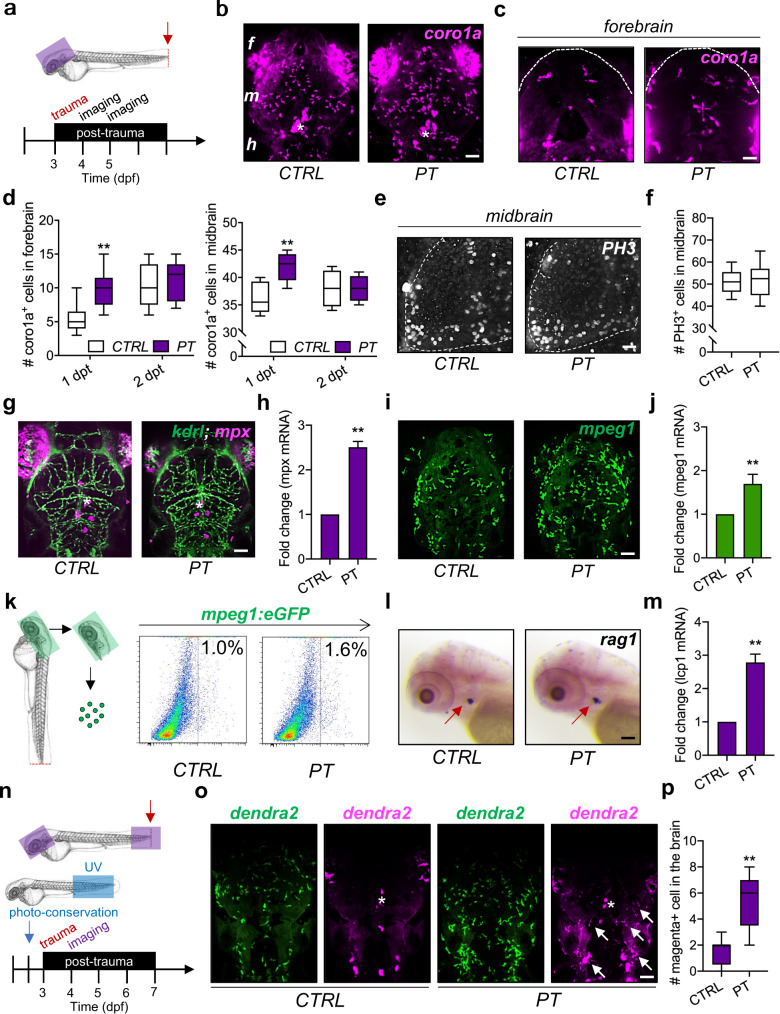
Leukocytes invade the brain after peripheral trauma in zebrafish larvae. **a** Experimental setup. dpf, day post-fertilization. **b–d** Light-sheet imaging showed a significantly increased number of *coro1a* + leukocytes in the zebrafish whole brain/midbrain and forebrain with peripheral trauma (PT) at 1 day post-trauma (dpt) but not at 2 dpt compared with the control (CTRL). Independent *t* test, **, *p* < 0.01. Scale bar, 40 µm (whole brain), 20 µm (forebrain). **f** forebrain. m, midbrain. h, hindbrain. Asterisk, autofluorescence of pigment. **e–f** Phospho-histone H3 (PH3) staining indicated no difference in the number of cells in mitosis in the brain between CTRL and PT. Independent *t* test. Scale bar, 20 µm. **g**, **h** qPCR but not light-sheet imaging showed significantly increased expression or the number of *mpx1* + neutrophils in the head at 1 dpt. Independent *t* test, **, *p* < 0.01. Scale bar, 40 µm. Asterisk, autofluorescence of pigment. **i–j** Light-sheet imaging and qPCR showed significantly increased expression or the number of *mepg1* + macrophages in the head at 1 dpt. Independent *t* test, **, *p* < 0.01. Scale bar, 40 µm. **k** Flow cytometry detected an increased number of *mepg1* + macrophages in the head after peripheral trauma. (**l**) No difference in *rag1* + lymphocytes (red arrow) was found in whole-mount in situ hybridization (WISH) between CTRL and PT. Scale bar, 40 µm. **m** qPCR showed significantly increased expression of *lcp1* + leukocytes in the head after peripheral trauma. Independent *t* test, **, *p* < 0.01. **n** Experimental setup for dendra2 photoconversion and peripheral leukocyte tracking. **o–p** A significantly elevated number of photoconverted magenta+ leukocytes (white arrow) in the brains of zebrafish after peripheral trauma. Independent *t* test, **, *p* < 0.01. Scale bar, 40 µm.

Zebrafish larvae with an evolutionarily conserved innate immune system have been used as a robust model of immune responses to peripheral trauma in the past decade^23^. Here, the amputation assay was performed on the tail of zebrafish, which is the farthest body region from the brain, at 3 dpf to model peripheral trauma in zebrafish larvae (Fig. 1a). The number of leukocytes labeled by *coro1a:DsRed* (*coro1a* + ), which largely consist of macrophages, neutrophils, and lymphocytes^23^, significantly increased in the midbrain and forebrain of transgenic zebrafish after peripheral trauma at 4 dpf/1 dpt, while the number of leukocytes returned to baseline compared to CTRL at 5 dpf/2 dpt (Fig. 1b–d). In addition, the number of PH3 + proliferating cells in the brains of 4-dpf zebrafish larvae remained unchanged after peripheral trauma (Fig. 1e–f). To distinguish the leukocyte subsets, *Tg(kdrl:GFP;mpx:mCherry)* and *Tg(mpeg1:GFP)* zebrafish lines were utilized to visualize *mpx* + neutrophils and *mpeg1* + macrophages in the brain after peripheral trauma, respectively (Fig. 1g–j). We found that the peripheral trauma-induced increase in leukocytes was largely due to *mpeg1* + macrophages, which was also confirmed by qPCR of *mpeg1* mRNA and flow cytometry of *mpeg1* + macrophages in the head (Fig. 1i–k). While the mRNA level of *mpx* was also elevated in the head of zebrafish larvae with peripheral trauma, no *mpx* + neutrophils were observed inside the brain (Fig. 1g–h), which suggested a failure of neutrophil invasion into the brain after recruitment to the head. Moreover, similar to *mpx* + neutrophils, no *rag1* + lymphocytes were found in the brains of zebrafish after peripheral trauma, although the number of increased *lcp1* + leukocytes was probably higher than that of *mpeg1* + macrophages in the head based on the fold change in mRNA (Fig. 1l–m). To further address whether these increased macrophages in the brain are peripherally derived, *mpeg1* + macrophages were tracked in various body regions at different time points using *Tg(mpeg1:GFP)* zebrafish. We found that the increased macrophages in the brain as well as the traumatic site were largely redistributed macrophages from the CHT and/or aorta-gonad-mesonephros (AGM), as the total number of macrophages remained unchanged after peripheral trauma, which was observed at 3 and 6 hours post-trauma (hpt) (Supplementary Fig. 1a–d). Furthermore, *Tg(mpeg1:dendra2)* zebrafish with photoconverted *dendra2* + macrophages from green to magenta in the tail region at 2.5 dpf were also utilized (Fig. 1n and Supplementary Fig. 2a-b). As expected, magenta+ macrophages were detected in both the brain and the traumatic site of 4-dpf zebrafish larvae after peripheral trauma (Fig. 1o and Supplementary Fig. 3). The number of magenta+ peripheral macrophages in the brain was significantly higher than that in the CTRL, although very few magenta+ macrophages (less than two) were observed in some of the CTRL zebrafish, which may be macrophages inside the cerebral vessel or on the skin (Fig. 1p). Collectively, these findings suggested that peripheral leukocytes, largely macrophages, transiently invaded the brain after a short period of peripheral trauma.

### Systemic inflammation mediates leukocyte invasion into the brain after peripheral trauma

**Fig. 2:**
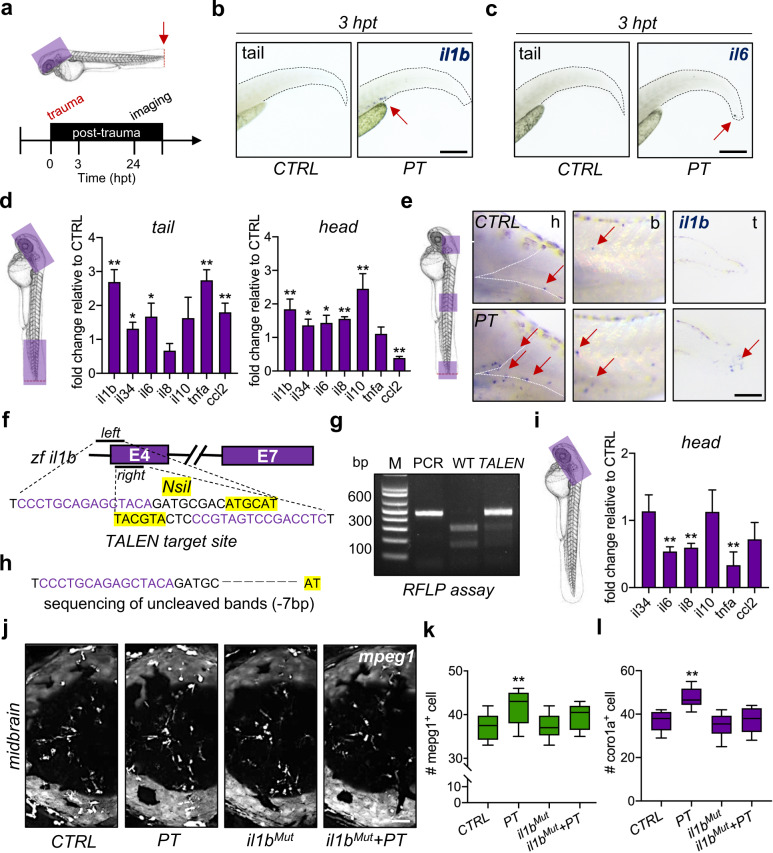
Systemic inflammation mediates leukocyte invasion into the brain after peripheral trauma. **a** Experimental setup. hpt, hours post-trauma. **b**, **c** The expression of *il1b* + and *il6* + cells (red arrow) in zebrafish larvae was measured by WISH at 3 hpt. Scale bar, 200 µm. **d** qPCR showed that the mRNA levels of various cytokines or chemokines changed in the traumatic site/tail and the brain/head compared to CTRL. Independent *t* test, *, *p* < 0.05, **, *p* < 0.01. **e** WISH showed increased *il1b* + cells (red arrow) in the brain/head, body/trunk, and traumatic site/tail of zebrafish larvae at 24 hpt. Scale bar, 100 µm. h, head. b, body. t, tail. **f** Design of the transcription activator-like effector nucleases (TALEN) targeting zebrafish *il1b*. **g**, **h** *il1b* mutation (*il1b*^*Mut*^) was confirmed by the restriction fragment length polymorphism (RFLP) assay and Sanger sequencing (−7 bp). (**i**) qPCR showed that the mRNA levels of various cytokines were decreased in *il1b*^*Mut*^ zebrafish brains compared to CTRL brains. Independent *t* test, **, *p* < 0.01. **j–l** *il1b*^*Mut*^ rescued the increased *mpeg1* + macrophages or *coro1a* + leukocytes in the zebrafish brain at 24 hpt. Two-way ANOVA with Tukey’s HSD post hoc test, **, *p* < 0.01 compared to CTRL. Scale bar, 40 µm.

Systemic inflammation is a common sign of peripheral trauma, in which proinflammatory cytokines increase immediately and circulate throughout the body, which can serve as the driver of leukocyte invasion into the brain^8^, while activated leukocytes are one of the sources of inflammatory cytokines. The levels of inflammatory cytokines were measured at 3 hpt and 24 hpt in the current study (Fig. 2a). Following peripheral trauma, *il1b* + cells, which are leukocytes as confirmed in a previous study^33^, were observed in the CHT of zebrafish at 3 hpt, while almost no *il6* + cells were detected by using WISH (Fig. 2b-c). On the other hand, elevated inflammatory cytokine/chemokine mRNA, including *il1b*, *il34*, *il6*, tumor necrosis factor-a *(tnfa)*, and C-C motif chemokine ligand 2 (*ccl2)* but not *il8* and *il10*, were detected in the tail/traumatic region at 4 dpf, indicating peripheral inflammation (Fig. 2d). More importantly, increased mRNA expression of *il1b*, *il34*, *il6*, *il8*, and *il10* but not *tnfa* and decreased mRNA expression of *ccl2* were detected in the head region at 4 dpf, suggesting peripheral trauma-induced neuroinflammation (Fig. 2d). Moreover, *il1b* + cells were observed throughout the body of zebrafish larvae after peripheral trauma, particularly in the tail and brain regions (Fig. 2e). This result implied a potential role of leukocytes in peripheral trauma-induced neuroinflammation or systemic inflammation. However, the role of systematic inflammation in leukocyte invasion of the brain after peripheral trauma remains unclear. Thus, an *il1b* mutant zebrafish line was established using TALEN (Fig. 2f–h). Somatic and stable *il1b* mutants displayed normal gross development and significantly decreased mRNA expression of major inflammatory cytokines, including *il6*, *il8*, and *tnfa*, but not *il34*, *il10*, and *ccl2*, in the head of zebrafish larvae (Fig. 2i). Importantly, attenuation of systemic inflammation by *il1b* mutation significantly blocked the increase in *coro1a* + leukocytes or *mpeg1* + macrophages in the brain after peripheral trauma, while *il1b* mutation alone showed no effect on the number of leukocytes in the brain (Fig. 2j–l). Therefore, leukocyte invasion of the brain after peripheral trauma is mediated by *il1b*-regulated systemic inflammation.

### Myeloid cells and systemic inflammation are involved in posttraumatic anxiety-like behaviors

**Fig. 3:**
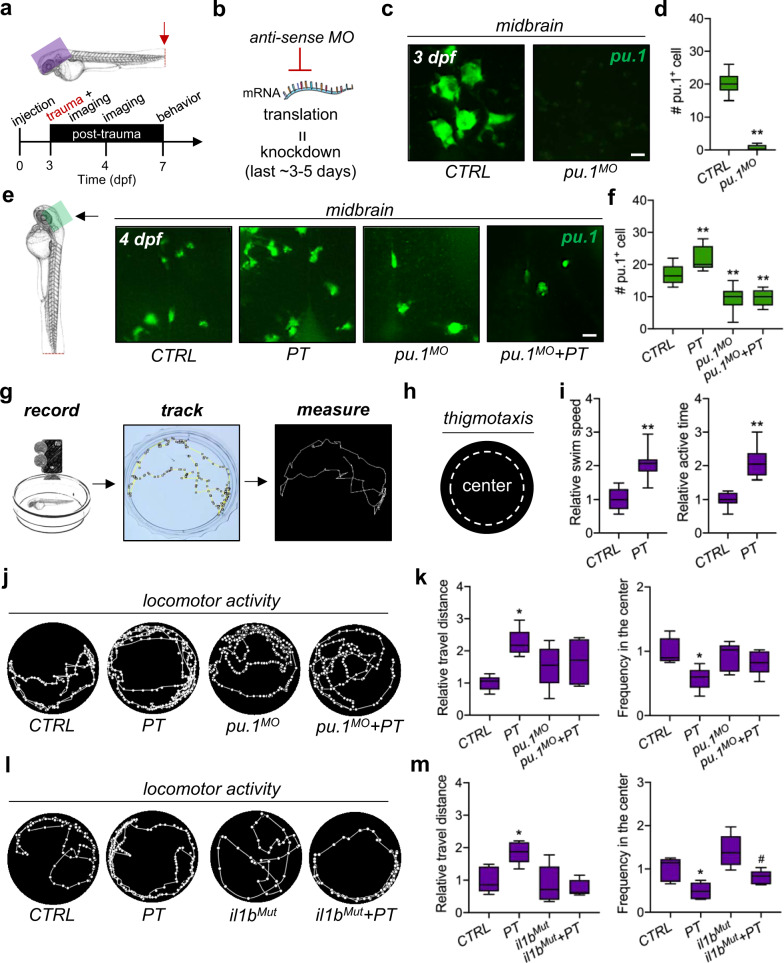
Myeloid cells and systemic inflammation are involved in posttraumatic anxiety-like behaviors. **a** Experimental setup. **b** Schematic showing antisense morpholino (MO)-mediated transient knockdown. **c**, **d** Light-sheet imaging showed a significantly decreased number of *pu.1* + myeloid cells by *pu.1* MO knockdown in the zebrafish brain at 3 dpf. Independent *t* test, **, *p* < 0.01. Scale bar, 10 µm. **e**, **f** Light-sheet imaging showed that *pu.1* MO knockdown inhibited the increase in *pu.1* + myeloid cells in the zebrafish brain at 1 dpt. Two-way ANOVA with Tukey’s HSD post hoc test, **, *p* < 0.01 compared with CTRL. Scale bar, 20 µm. **g**, **h** Schematic showing the locomotor behavioral assay and thigmotaxis for zebrafish larvae. **i** Peripheral trauma increased the swim speed and active time of zebrafish larvae at 7 dpf/4 dpt. Independent *t* test, **, *p* < 0.01. **j–k** Behavioral assays suggested anxiety-like behaviors, including hyperactivity and thigmotaxis, in zebrafish at 4 dpi, which could be rescued by *pu.1* MO knockdown. Two-way ANOVA with Tukey’s HSD post hoc test, *, *p* < 0.05 compared with CTRL. **l–m** *il1b* mutation rescued hyperactivity but not thigmotaxis at 4 dpi. Two-way ANOVA with Tukey’s HSD post hoc test, *, *p* < 0.05 compared to CTRL, #, *p* < 0.05 compared to *il1b*^*Mut*^.

To investigate the potential impact of systemic inflammation and myeloid cells on the brain, a behavioral assay was applied to zebrafish larvae after recovery from peripheral trauma (Fig. 3a). First, transient blockage of myeloid cells, largely microglia and invading macrophages in the brain, was conducted by *pu.1* MO knockdown that resulted in the loss of *pu.1* + myeloid cells in the brains of zebrafish at 3 dpf (Fig. 3b-d). Since the knockdown effect of morpholino generally lasts up to 5 days^34^, decreased *pu.1* + myeloid cells were still detected at 4 dpf, and transient *pu.1* MO knockdown inhibited the transiently increased macrophages at 4 dpf (Fig. 3e–f). The myeloid cells in the brain, particularly resident microglia, will recover after 5 dpf; transient *pu.1* knockdown thus primarily blocks the transient macrophage invasion and reactions of macrophages and microglia in response to peripheral trauma. Second, locomotor activity and thigmotaxis assays were performed in 7-dpf zebrafish larvae after recovery (Fig. 3g–h). Increased swim speed, time spent swimming (active time), and travel distance were found in the zebrafish that experienced peripheral trauma (Fig. 3i–k), suggesting hyperactive behaviors (restlessness) but not pain-related behaviors, as pain reduces swimming activities in zebrafish^35^. In addition, thigmotaxis (avoidance), a behavior with a tendency to remain close to the walls, was also observed (Fig. 3j–k), which, together with hyperactive behaviors (restlessness), are common indices of anxiety in both human and animal models^29^. Thus, peripheral trauma induced posttraumatic anxiety-like behaviors in zebrafish larvae. More importantly, blockage of transient leukocyte invasion by *pu.1* MO rescued these anxiety-like behaviors (Fig. 3j–1k), which indicated that macrophage invasion in the brain might drive the development of anxiety-like behaviors in zebrafish larvae after peripheral trauma. While the transient loss of macrophages and microglia showed no effects on the hyperactive behaviors and thigmotaxis in CTRL, their inflammatory reactions after peripheral trauma may also contribute to posttraumatic anxiety-like behaviors. Unlike transient *pu.1* knockdown, *il1b*^*Mut*^ rescued hyperactive behaviors, including the increased total travel distance, active time, and swim speed after peripheral trauma, but it failed to rescue thigmotaxis (Fig. 3l–m). Therefore, attenuation of systemic inflammation can only partially rescue anxiety-like behaviors after peripheral trauma, which suggests an *il1b*-independent role of myeloid cells in posttraumatic neurological disorders, and *il1b*-mediated leukocyte invasion of the brain is probably correlated with hyperactive behaviors after peripheral trauma.

### Leukocytes invade the zebrafish brain through vasculature-independent migration

**Fig. 4:**
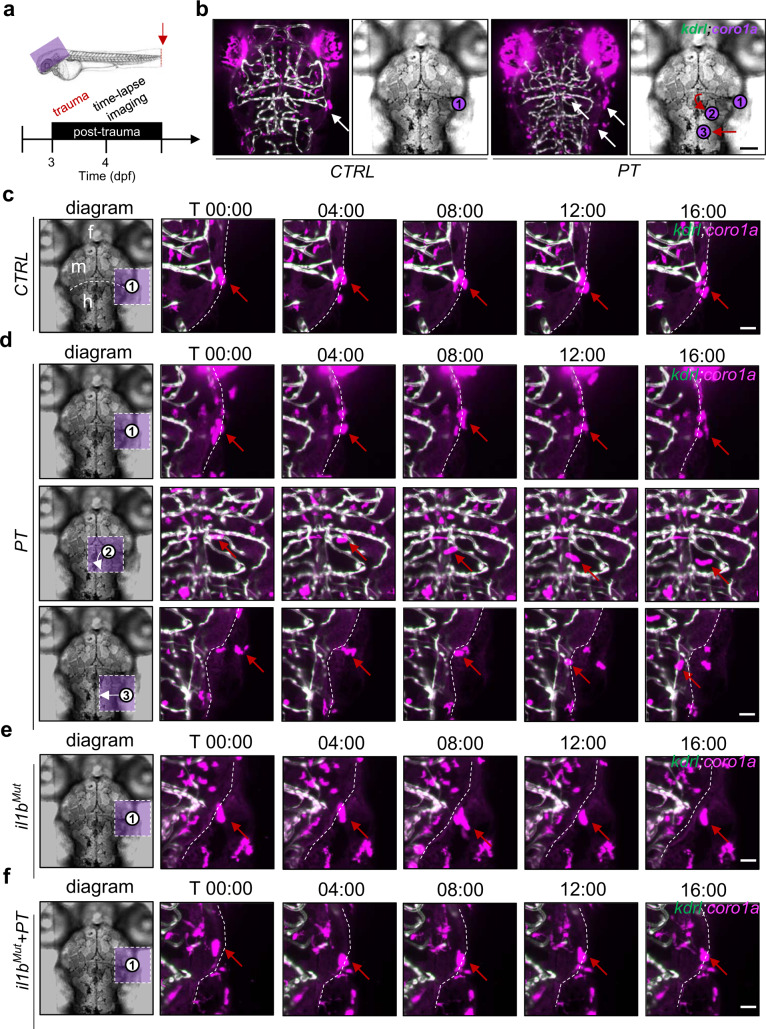
Leukocytes invade the zebrafish brain through vasculature-independent migration. **a** Experimental setup. **b** Active leukocytes (white arrow) were distributed in the brains of zebrafish with or without peripheral trauma. Scale bar, 40 µm. **c** Time-lapse light-sheet imaging showed the active leukocytes (red arrow) around the zebrafish brain in CTRL. Scale bar, 20 µm. **d** Time-lapse light-sheet imaging showed active leukocytes (red arrow) around the brain (1), inside the brain (2), and invading the brain (3) at 4 dpf. Scale bar, 20 µm. **e** Time-lapse light-sheet imaging showed the active leukocytes (red arrow) around the brain in *il1b*^*Mut*^ zebrafish. Scale bar, 20 µm. **f** Time-lapse light-sheet imaging showed the active leukocytes (red arrow) around the brain in *il1b*^*Mut*^ zebrafish at 4 dpf. Scale bar, 20 µm.

Time-lapse imaging was used to investigate the migration routes of systemic inflammation-mediated leukocyte invasion into the brain at 4 dpf when the microglia ceased to invade the brain during development^30^ (Fig. 4a). By using the *Tg(kdrl:GFP;coro1a:DsRed)* double transgenic zebrafish line to visualize cerebral blood vessels (*kdrl* + endothelial cells) and leukocytes, active leukocytes were observed in the head of the zebrafish after peripheral trauma while leukocytes in the CTRL zebrafish were relatively static (Fig. 4b). More importantly, the vasculature-independent migration of leukocytes into the brain was observed at 4 dpf, while no leukocytes were found to move in or out of the bloodstream or blood vessels in the current study. A schematic diagram in the left panel shows the distribution of active leukocytes around the brain (Fig. 4c–f). Specifically, peripheral leukocytes located in the neighboring tissues invaded the brain through the lateral periphery of the hindbrain after peripheral trauma (Fig. 4d and Supplementary Video 1). Conversely, such invasion could not be seen in the CTRL, *il1b*^*Mut*^, and *il1b*^*Mut*^ with peripheral trauma groups (Fig. 4c, 4e–f, and Supplementary Video 2–4). Active leukocytes distributed around the mid-hindbrain boundary (MHB) failed to invade the brain of 4-dpf zebrafish with or without peripheral trauma (Fig. 4c–f and Supplementary Video 5). In addition, compared to the CTRL, *il1b*^*Mut*^, and *il1b*^*Mut*^ with peripheral trauma groups, more active leukocytes were found within the brain after peripheral trauma (Fig. 4c–f and Supplementary Video 6), which might be due to the early invasion of leukocytes through the lateral periphery of the hindbrain (Fig. 4d and Supplementary Video 7). Therefore, these results demonstrated that peripheral trauma induced systemic inflammation-regulated invasion of leukocytes into the brain from neighboring tissues through the lateral periphery of the hindbrain.

### Peripheral leukocytes are recruited by systemic inflammation-induced apoptosis in the brain

**Fig. 5:**
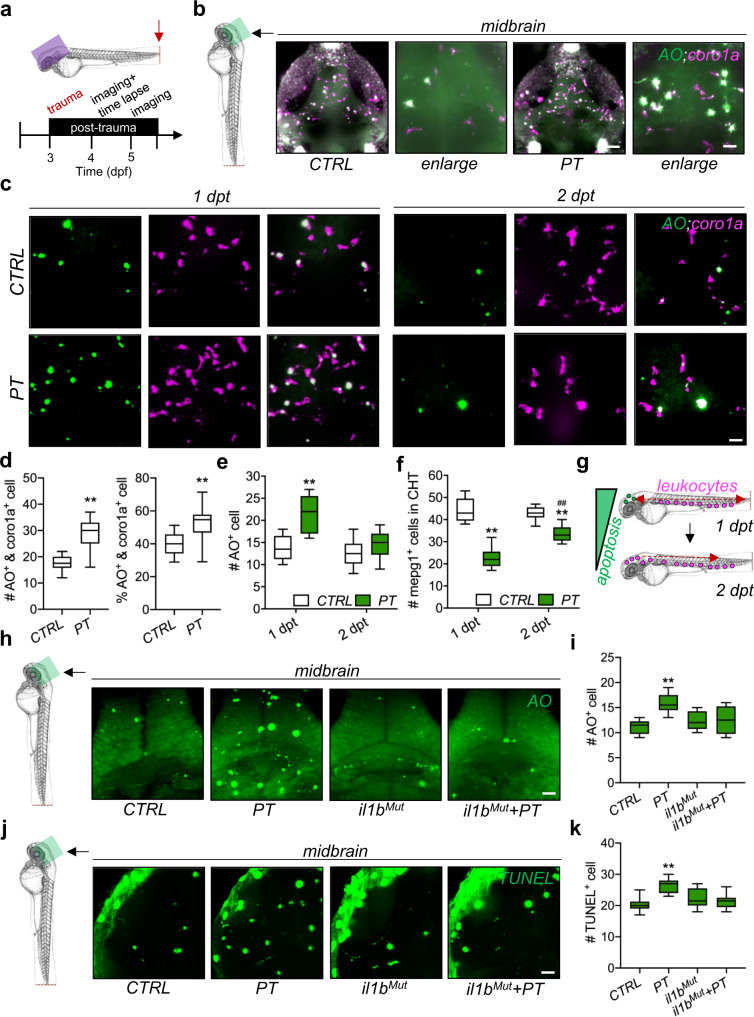
Invading leukocytes are recruited for the clearance of apoptotic cells in the brain. **a** Experimental setup. **b, d** Light-sheet imaging showed an increased number and percentage of the colocalization of *coro1a* + leukocytes and AO + apoptotic cells in the zebrafish brain at 4 dpt. Independent *t* test, **, *p* < 0.01 compared with CTRL. **c, e** Light-sh**e**et imaging showed an increased number of AO + apoptotic cells in the zebrafish brain at 1 dpt but not at 2 dpt. Independent *t* test, **, *p* < 0.01 compared to CTRL. **f** Light-sheet imaging showed that *mpeg1* + macrophages returned to the CHT at 2 dpt. Two-way ANOVA with Tukey’s HSD post hoc test, **, *p* < 0.01 compared to CTRL at 1 dpt, #, *p* < 0.05 compared to CTRL at 2 dpt. Scale bar, 5 µm. **g** Schematic showing that apoptosis in the brain is associated with leukocyte distribution. **h–k** *il1b* mutation rescued the increased number of AO + or TUNEL + apoptotic cells in the zebrafish brain at 1 dpi. Two-way ANOVA with Tukey’s HSD post hoc test, **, *p* < 0.01 compared to CTRL. Scale bar, 40 µm.

**Fig. 6:**
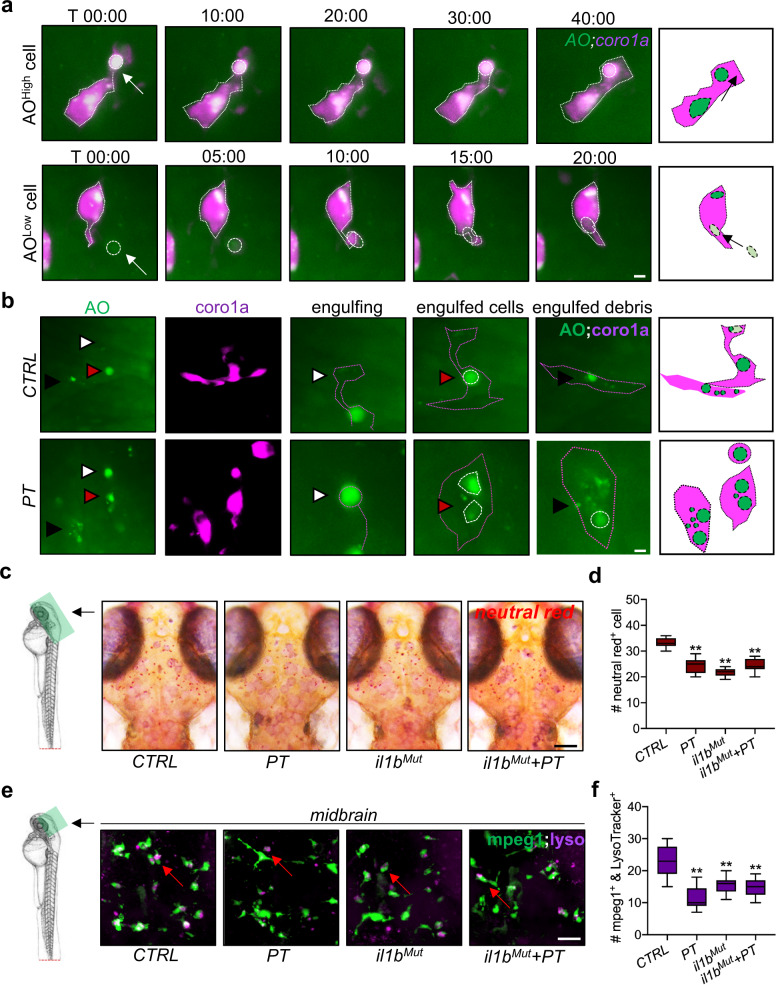
Microglial lysosome impairment may account for the leukocyte invasion of the brain. **a**, **b** Time-lapse light-sheet imaging showed the phagocytosis of AO^high^ and AO^low^ cells (white arrow or arrowhead) by *coro1a* + leukocytes in the zebrafish brain at 1 dpt. **c**, **d** Neutral red staining showed microglial lysosome impairment in zebrafish brains with or without *il1b* mutation at 1 dpt. Two-way ANOVA with Tukey’s HSD post hoc test, **, *p* < 0.01 compared to CTRL. Scale bar, 40 µm. **e**, **f** LysoTracker staining showed *mpeg1* + microglial lysosome impairment in the zebrafish brain at 1 dpt or under *il1b* mutation. Two-way ANOVA with Tukey’s HSD post hoc test, **, *p* < 0.01 compared to CTRL. Scale bar, 20 µm.

Aberrant accumulation of apoptotic cells in the brain is a trigger of peripheral leukocyte invasion of the brain for the clearance of dead cells^36^. Thus, the interplay between apoptotic cells and leukocytes was also examined in the zebrafish brain after peripheral trauma (Fig. 5a). Elevated *coro1a* + and AO + (apoptotic marker) colocalized signals and an increased percentage of AO + leukocytes (divided by the total number of *coro1a* + cells) were observed in the brain after peripheral trauma (Fig. 5b and d). In accordance with the transient invasion of leukocytes in the brain, increased apoptotic cells in the brain after peripheral trauma returned to baseline at 5 dpf/2 dpt (Fig. 5c and e), while some of the macrophages also migrated back to the CHT at 2 dpt (Fig. 5f and Supplementary Fig. 4a–b), which illustrated that invading leukocytes may clean up the aberrant apoptotic cells before they leave the brain and that apoptosis signals in the brain may recruit the peripheral leukocytes to the brain (Fig. 5g). In addition, the accumulation of apoptotic cells labeled by AO and TUNEL staining in the brain was induced by systemic inflammation after peripheral trauma since they can be alleviated by the attenuation of systemic inflammation (Fig. 5h–k). Therefore, apoptotic cells in the brain are potentially induced by peripheral trauma-induced systemic inflammation. To determine the interplay among neuroinflammation, apoptosis, and leukocytes in the brain during leukocyte recruitment after peripheral trauma, qPCR was performed to monitor the changes in *il1b*, *caspase3*, and *lcp1* mRNA in the brain. We found that *il1b* in the brain increased shortly after peripheral trauma and peaked at approximately 1 hpt, while significantly increased *caspase3* and *lcp1* were observed from approximately 1 hpt and 2 hpt, respectively (Supplementary Fig. 5a–b). These findings supported that leukocytes were potentially recruited by systemic inflammation-induced apoptosis, while they may influence each other after leukocyte invasion. On the other hand, time-lapse imaging showed that an AO + apoptotic cell was phagocytized by leukocytes in the brain; however, a cell with low-level AO signal was also phagocytized (Fig. 6a). Additionally, more AO + cells and cell debris were found in the leukocytes in the brain after peripheral trauma than in the CTRL (Fig. 6b). Therefore, microglia and invading leukocytes may be more phagocytic in response to peripheral trauma and remove nonapoptotic cells, consequently resulting in brain damage. On the other hand, the elevated apoptosis signal in the brain can be partially attributed to the impaired degradation in microglia, as neutral red+ or LysoTracker+ lysosomes in microglia decreased after peripheral trauma (Fig. 6c–f). Interestingly, *il1b* mutation alone also impaired normal phagocytic activities of microglia, which was characterized by reduced lysosomes within microglia (Fig. 6c–f). However, unlike peripheral trauma, *il1b* mutation-induced phagocytic dysfunction of microglia did not lead to the accumulation of apoptotic cells in the brains of zebrafish embryos (Fig. 5h–k). This result indicated that phagocytic impairment of microglia was not the main trigger of elevated apoptosis in the brain, although it may exacerbate the accumulation of apoptotic cells induced by peripheral trauma due to a failure in housekeeping. In addition, a proper level of *il1b* was required for microglial phagocytosis in the brains of zebrafish embryos. These results demonstrated that peripheral trauma-induced lysosome impairment is probably regulated by *il1b*, which, however, is required for normal phagocytosis, and more importantly, *il1b*-mediated systemic inflammation is the major driver of apoptosis in the brain after peripheral trauma.

### Systemic inflammation-mediated proteomic responses of the brain to peripheral trauma

**Fig. 7:**
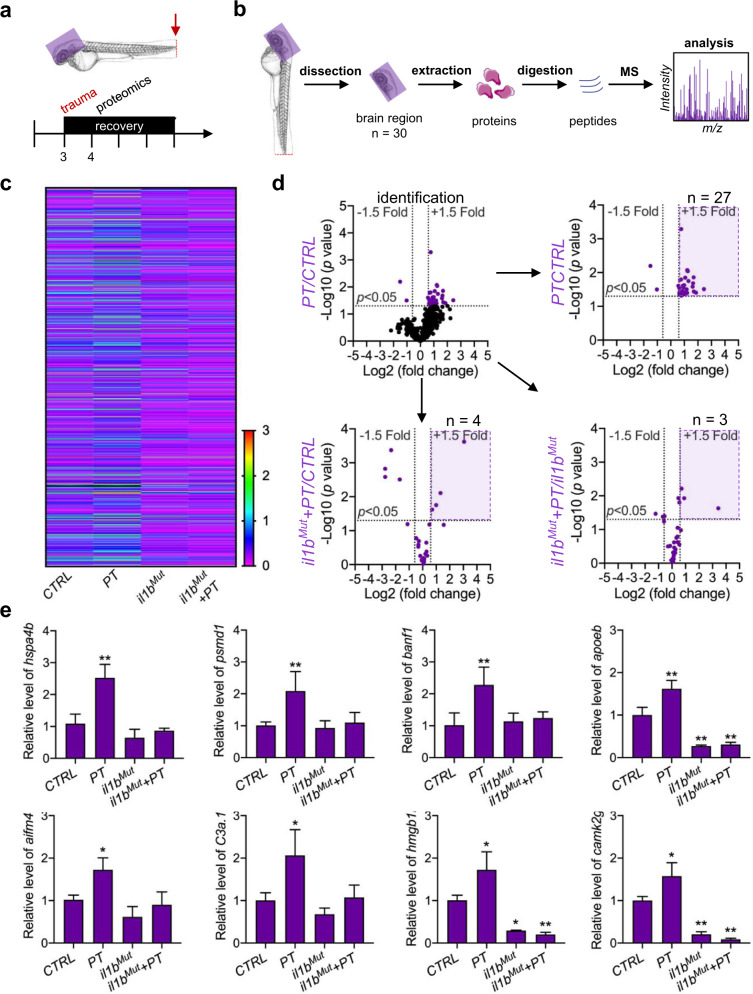
Systemic inflammation-mediated proteomic responses in the brain to peripheral trauma. **a** Experimental setup. **b** Schematic showing the proteomic analysis in the head region of zebrafish larvae after peripheral trauma. MS, mass spectrometry. **c** Heatmap illustrating the fold change in proteins among the CTRL, PT, *il1b*^*Mut*^, and *il1b*^*Mut*^ + PT groups. **d** The volcano plot illustrates the proteins that were significantly (*p* < 0.05) changed (>1.5-fold) in the head region of zebrafish after peripheral trauma and the change in these identified proteins (purple) in various conditions: (1) PT/CTRL, (2) *il1b*^*Mut*^ + PT/CTRL, and (3) *il1b*^*Mut*^ + PT/*il1b*^*Mut*^. n, the number of identified proteins that significantly increased. **e** The relative levels of proteins in the CTRL, CTRL + PT, *il1b*^*Mut*^, and *il1b*^*Mut*^ + PT groups. Two-way ANOVA with Tukey’s HSD post hoc test, *, *p* < 0.05, **, *p* < 0.01 compared with CTRL.

To further investigate the molecular mechanisms underlying systemic inflammation-mediated leukocyte invasion of the brain after peripheral trauma, quantitative proteomics was performed on the brain regions of zebrafish embryos (Fig. 7a–b). Following protein identification and quantification, 29 out of 803 (3.6%) proteins were found in the head that showed significant changes after peripheral trauma, which included 27 increased proteins and two decreased proteins (Fig. 7c–d). We tracked these 29 proteins in various proteomic analyses under *il1b* mutation with or without peripheral trauma and excluded proteins that did not respond to peripheral trauma (Fig. 7d and Supplementary Fig. 6a–c). Finally, 21 out of 29 (72%) proteins were found in the brain that were regulated by systemic inflammation after peripheral trauma, with the expression alleviated in *il1b*^*Mut*^ (Supplementary Fig. 6d). The function of these proteins was further classified to explore the effect of peripheral trauma-induced systemic inflammation on the brain (Supplementary Fig. 6e). In accordance with previous findings, *apoptosis inducing factor mitochondria associated 4* (*aifm4*) and *apolipoprotein Eb* (*apoeb*), specific markers of pro-apoptosis and phagocytosis, were found to be regulated by peripheral trauma-induced systemic inflammation. Proteins involved in inflammation (*complement C3a, tandem duplicate 1* [*c3a.1*], *CaM kinase II gamma 1* [*camk2g1*], *high mobility group box 1b* [*hmgb1b*], *heat shock protein 4b* [*hspa4b*], and *proteasome 26* *S subunit, non-ATPase 1* [*psmd1*]), neuroprotection (*BAF nuclear assembly factor 1* [*banf1*], *cold-inducible RNA-binding protein* [*cirpb*], and *protein kinase, cAMP-dependent, regulatory, type II, alpha A* [*prkar2aa*]), and metabolism (*ATPase Na* + */K* + *transporting subunit alpha 3a* [*atp1a3a*], *ATPase Na* + */K* + *transporting subunit beta 4* [*atp1b4*], *phosphofructokinase 1b* [*pfk1b*], and *oxoglutarate dehydrogenase* [*ogdh*]) were also identified (Fig. 7e). Importantly, *camk2g1* and *hmgb1b*, orthologous to human CAMK2G and HMGB1, respectively, were detected, which are implicated in posttraumatic neurological disorders (Fig. 7e)^37,38^. These findings supported that peripheral trauma in zebrafish larvae represents a translational model of peripheral trauma-induced neurological disorders in humans, which is mainly regulated by systemic inflammation. Moreover, *c3a.1*, orthologous to human C3a, which is a potential mediator together with *aifm4* for leukocyte recruitment to the brain during systemic inflammation, was elevated in the brain (Fig. 7e)^39^ and may be the key regulator of leukocyte invasion into the brain during systemic inflammation after peripheral trauma.

## Discussion

**Fig. 8:**
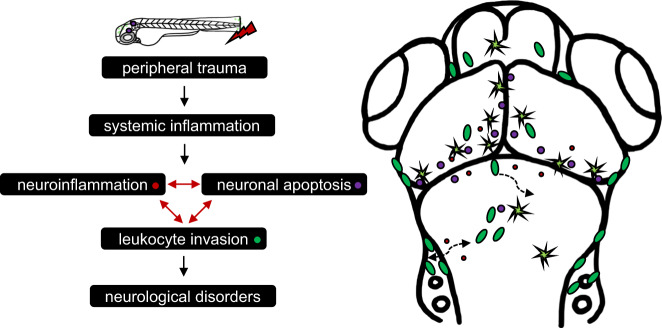
Summary of systemic inflammation-mediated brain responses to peripehral trauma. Schematic diagram of systemic inflammation-mediated leukocyte invasion and its impacts on the brain after peripheral trauma.

Peripheral leukocyte invasion of the brain is a hallmark of many neurological disorders in peripheral inflammatory diseases^8–11^. However, its roles in neurological disorders that occur after peripheral trauma remain largely unknown. In this study, we employed transgenic zebrafish models to investigate systemic inflammation and leukocyte trafficking in response to peripheral trauma and their potential influences on the CNS, which is potentially associated with the development of posttraumatic neurological disorders. Our findings demonstrated that peripheral leukocytes invaded the brain during systemic inflammation after peripheral trauma and played complex roles in the brain, which involved an interplay among invading leukocytes, neuroinflammation, and neuronal apoptosis through *il1b*-regulated mechanisms (Fig. 8). To the best of our knowledge, this is the first study to report the observation of leukocyte invasion into the brain after peripheral trauma in an animal model, and it provided evidence that leukocyte invasion of the brain during systemic inflammation is the potential mechanism behind the etiology of posttraumatic neurological disorders, while further studies are still needed.

Our previous studies have revealed that systemic inflammation-induced neuroinflammation facilitates the development of multiple neurological disorders, in which proinflammatory cytokines, largely IL-1β, IL-6, and TNF-α, are increased in the CNS accompanied by oxidative stress, apoptosis, and tau pathogenesis^20,40,41^. These elevated proinflammatory cytokines in the CNS can result from circulating cytokines from the periphery and activated leukocytes in chronic inflammation. Over the past two decades, evidence has accumulated that leukocytes are able to invade the brain and play important roles in the pathology of trauma-induced or stress-induced neurological disorders during systemic inflammation^42,43^, which also serves as the mechanism that underlies neurological disorders induced by various peripheral inflammatory diseases. Although peripheral trauma has long been characterized by systemic inflammatory reactions, such as SIRS, the impacts of circulating cytokines and peripherally derived leukocytes on the CNS after peripheral trauma remain unclear^14^. Our work provided experimental evidence of peripheral leukocyte invasion into the brain during systemic inflammation after peripheral trauma using a transgenic zebrafish model and demonstrated the complex impacts of invading leukocytes on the CNS through *il1b*-regulated mechanisms, which provided a basis to stimulate the understanding of neurological disorders that occur after peripheral trauma and the development of novel therapeutic strategies by targeting leukocyte invasion.

Although leukocyte invasion into the brain is featured in multiple neurological diseases, the precise roles of invading leukocytes in the pathogenesis of these diseases remain elusive^5,44^. Commonly, invading leukocytes serve as contributing factors to the development of neurological disorders, although they can also protect the CNS in a stress-type-specific manner^45,46^. It has been documented that disturbances may occur shortly after trauma in stress-responsive brain regions, such as the hippocampus, amygdala, and prefrontal cortex, which are responsible for the development of posttraumatic neurological disorders^47^. For instance, stress-induced transient leukocyte invasion in the hippocampus, amygdala, and prefrontal cortex of the brain drives anxiety-like behaviors in mice^44^. Similarly, we also found that leukocytes, largely macrophages, transiently invaded the brain of zebrafish after peripheral trauma and were located in the hindbrain, midbrain, and forebrain, which are the conserved brain regions that correspond to the hippocampus, amygdala, and prefrontal cortex in mammals^48^. The distribution of invading leukocytes in these specific brain regions implicates that posttraumatic neurological disorders develop due to interference with the steady state of the brain^44^. Therefore, the invasion and distribution of peripheral leukocytes in the brain are associated with posttraumatic anxiety-like behaviors in zebrafish. Furthermore, blockade of leukocyte invasion of the brain by transient *pu.1* knockdown or *il1b* mutation rescued anxiety-like behaviors, particularly hyperactive behavior, in zebrafish larvae, which confirmed that invading leukocytes during systemic inflammation are potentially involved in the development of peripheral trauma-induced neurological disorders, while their precise roles require further study.

On the other hand, systemic inflammation-induced neuroinflammation was identified after peripheral trauma and is implicated in the pathology of neurological disorders in many peripheral inflammatory diseases^8–10^. Interestingly, invading leukocytes can serve as carriers or sources of proinflammatory cytokines in the brain during systemic inflammation, while they can also be recruited by neuroinflammation. In the current study, *il1b-*expressing cells were detected simultaneously with elevated inflammatory cytokines at both the traumatic site and in the brain of zebrafish larvae after peripheral trauma, whereas attenuation of systemic inflammation by *il1b* knockout suppressed leukocyte invasion. While CCL2 and TNF-α are known as chemoattractant or cytoattractant stimuli for leukocyte recruitment to the brain in various pathological conditions^8,49,50^, their levels remained unchanged or decreased in the brain after peripheral trauma in the present study. However, we revealed that IL-1β is a cytoattractant stimulus for leukocyte invasion of the brain after peripheral trauma, which coincides with the recruitment of leukocytes to the brain in repeat stress-induced anxiety^44^. These findings demonstrated reciprocal causation between neuroinflammation and leukocyte invasion of the brain after peripheral trauma, which may exacerbate neurological disorders, and the mechanism that underlies peripheral leukocyte invasion of the brain is disease specific. More importantly, we found that attenuation of systemic inflammation can only rescue hyperactive behavior but not thigmotaxis, while deletion of myeloid cells can recuse both of these behaviors after peripheral trauma. These findings indicated a potential *il1b*-independent role of resident microglia and invading macrophages in thigmotaxis after peripheral trauma in accordance with a previous study in which microglial deletion rescued thigmotaxis but not hyperactive behavior during PTSD^51^. This finding suggests that *il1b*-mediated leukocyte invasion of the brain is one of the mechanisms behind the neurological disorders resulting from peripheral trauma, particularly hyperactive behavior (restlessness); further studies on the unknown pathogenesis are still needed.

In addition, it is widely believed that peripheral leukocyte invasion of the brain requires BBB breakdown, although invading leukocytes are found in the CNS with an intact BBB and, in some cases, migrate through the cerebrospinal fluid^52,53^. In the current study, vasculature-independent cell migration was observed, where leukocytes invaded the brain from the neighboring tissues through the lateral periphery of the hindbrain, specifically the boundary or crevice between the metencephalon (or cerebellum) and myelencephalon. Leakage of leukocytes from the circulation was not observed, which suggested an intact and functional BBB for circulatory leukocytes in zebrafish larvae from 3 dpf^54^. Notably, the invasion route from the periphery to the brain differed from microglial invasion of the zebrafish midbrain during development^36^. This information suggests that the less studied vasculature-independent migration routes might be involved in the invasion of leukocytes into the brain under disease states, which can be distinct from their migration in physiological conditions. Activated leukocytes are capable of migrating within organs or tissues during immune surveillance in addition to trafficking through the bloodstream^55^. In the present study, the zebrafish model, as a robust in vivo cell tracking platform, provided novel information about leukocyte invasion into the brain during systemic inflammation after peripheral trauma, which is technically difficult to obtain in humans and rodent models, while more evidence on leukocyte invasion of the brain in mammalian models is necessary before translation from the laboratory to the clinic.

Furthermore, we also explored the impacts of invading leukocytes as well as neuroinflammation on the brain, which may contribute to the development of neurological disorders after peripheral trauma. Our results identified an *il1b*-mediated accumulation of apoptotic cells in the brain after peripheral trauma, which suggested *il1b*-induced neuronal damage as described in a previous study^21^. These apoptotic cells in the brain have long been recognized as the eat-me or find-me signal for peripheral leukocytes^36^. We also found that apoptotic cells were phagocytized by leukocytes in the zebrafish brain, which also transiently increased after peripheral trauma, although some nonapoptotic cells were also removed. The qPCR results showed that inflammation, apoptosis, and leukocytes were sequentially elevated in the brain shortly after peripheral trauma, in which a cytokine peak was observed before the increase in apoptosis and leukocytes in accordance with a previous study in TBI^56^. Therefore, the observation suggested that the *il1b*-induced accumulation of apoptotic cells is also an attractant for leukocyte invasion of the brain after peripheral trauma, wherein they were recruited to assist functionally impaired microglia in the removal of apoptotic cells from the brain^57^. These invading leukocytes, together with neuroinflammation, at least partially account for the brain damage after peripheral trauma and the subsequent neurological disorders. On the other hand, mass spectrometry-based proteomics was employed to investigate brain responses, especially *il1b*-mediated responses, to peripheral trauma. Intriguingly, *camk2g* and *hmgb1b*, the orthologs of human CaMK2G and HMGB1, are inflammation-regulated proteins that are increased in the brains of patients with posttraumatic neurological disorders^37,38^. In particular, HMGB1 has been found to be required for systemic inflammation-induced remote organ injury^55^. These similar brain proteomic responses between zebrafish and humans/rodent models suggested that zebrafish are a good translational model of neurological disorders after peripheral trauma. Additionally, we found a category of proteomic responses that are associated with apoptosis, inflammation, phagocytosis, and neuroprotection, including *aifm4*, *apobeb*, *banf1*, *cirpb*, *hspa4b*, and *psmd1*, which was consistent with the findings from other assays in the current study. Importantly, *C3a.1*, the ortholog protein of human C3a that was found to be responsible for leukocyte recruitment to the brain during inflammation^39^, was detected, which is a potential mediator between *il1b*-regulated systemic inflammation and leukocyte invasion of the brain after peripheral trauma. Thus, the regulatory role of *C3a.1* in leukocyte invasion of the brain during systemic inflammation will be explored in our future work, which may provide further information about the mechanisms underlying leukocyte invasion of the brain during systemic inflammation in neurological disorders after peripheral trauma.

## Supplementary information

Supplementary figures

Video 1.

Video 2.

Video 3.

Video 4.

Video 5.

Video 6.

Video 7.

## References

[1] Prinz M, Priller J (2017). The role of peripheral immune cells in the CNS in steady state and disease. Nat. Neurosci..

[2] Baruch K, Kertser A, Porat Z, Schwartz M (2015). Cerebral nitric oxide represses choroid plexus NFκB-dependent gateway activity for leukocyte trafficking. EMBO J..

[3] Möhle L (2016). Ly6C(hi) monocytes provide a link between antibiotic-induced changes in gut microbiota and adult hippocampal neurogenesis. Cell Rep..

[4] Prinz M, Priller J, Sisodia SS, Ransohoff RM (2011). Heterogeneity of CNS myeloid cells and their roles in neurodegeneration. Nat. Neurosci..

[5] Greenhalgh AD (2018). Peripherally derived macrophages modulate microglial function to reduce inflammation after CNS injury. PLoS Biol..

[6] Wohleb ES, Franklin T, Iwata M, Duman RS (2016). Integrating neuroimmune systems in the neurobiology of depression. Nat. Rev. Neurosci..

[7] Cao W, Zheng H (2018). Peripheral immune system in aging and Alzheimer’s disease. Mol. Neurodegener..

[8] D’Mello C, Le T, Swain MG (2009). Cerebral microglia recruit monocytes into the brain in response to tumor necrosis factoralpha signaling during peripheral organ inflammation. J. Neurosci..

[9] Buckman LB (2014). Obesity induced by a high-fat diet is associated with increased immune cell entry into the central nervous system. Brain. Behav. Immun..

[10] Garré JM, Silva HM, Lafaille JJ, Yang G (2017). CX3CR1(+) monocytes modulate learning and learning-dependent dendritic spine remodeling via TNF-α. Nat. Med..

[11] Zhu H, Liu W, Fang H (2018). Inflammation caused by peripheral immune cells across into injured mouse blood brain barrier can worsen postoperative cognitive dysfunction induced by isoflurane. BMC Cell Biol..

[12] MacKenzie EJ (2000). Epidemiology of injuries: current trends and future challenges. Epidemiol. Rev..

[13] Kilpatrick DG (2013). National estimates of exposure to traumatic events and PTSD prevalence using DSM-IV and DSM-5 criteria. J. Trauma. Stress.

[14] Lord JM (2014). The systemic immune response to trauma: an overview of pathophysiology and treatment. Lancet.

[15] Alam A, Hana Z, Jin Z, Suen KC, Ma D (2018). Surgery, neuroinflammation and cognitive impairment. EBioMedicine.

[16] Das M, Mohapatra S, Mohapatra SS (2012). New perspectives on central and peripheral immune responses to acute traumatic brain injury. J. Neuroinflammation.

[17] Brøchner AC, Toft P (2009). Pathophysiology of the systemic inflammatory response after major accidental trauma. Scand. J. Trauma Resusc. Emerg. Med..

[18] Nourshargh S, Alon R (2014). Leukocyte migration into inflamed tissues. Immunity.

[19] Lee JW (2008). Neuro-inflammation induced by lipopolysaccharide causes cognitive impairment through enhancement of beta-amyloid generation. J. Neuroinflammation.

[20] Huang C, Irwin MG, Wong GTC, Chang RCC (2018). Evidence of the impact of systemic inflammation on neuroinflammation from a non-bacterial endotoxin animal model. J. Neuroinflammation.

[21] Skelly DT (2019). Acute transient cognitive dysfunction and acute brain injury induced by systemic inflammation occur by dissociable IL-1-dependent mechanisms. Mol. Psychiatry.

[22] Fidalgo AR (2011). Systemic inflammation enhances surgery-induced cognitive dysfunction in mice. Neurosci. Lett..

[23] Yan B (2014). IL-1β and reactive oxygen species differentially regulate neutrophil directional migration and Basal random motility in a zebrafish injury-induced inflammation model. J. Immunol..

[24] Fontana BD, Mezzomo NJ, Kalueff AV, Rosemberg DB (2018). The developing utility of zebrafish models of neurological and neuropsychiatric disorders: a critical review. Exp. Neurol..

[25] Kimmel CB, Ballard WW, Kimmel SR, Ullmann B, Schilling TF (1995). Stages of embryonic development of the zebrafish. Dev. Dyn..

[26] Pei W (2016). Extracellular HSP60 triggers tissue regeneration and wound healing by regulating inflammation and cell proliferation. NPJ Regen. Med..

[27] Chen XK, Kwan JS, Chang RC, Ma AC (2020). 1-phenyl 2-thiourea (PTU) activates autophagy in zebrafish embryos. Autophagy.

[28] Sztal TE, Ruparelia AA, Williams C, Bryson-Richardson RJ (2016). Using touch-evoked response and locomotion assays to assess muscle performance and function in zebrafish. J. Vis. Exp..

[29] Kedra M (2020). TrkB hyperactivity contributes to brain dysconnectivity, epileptogenesis, and anxiety in zebrafish model of Tuberous Sclerosis Complex. Proc. Natl Acad. Sci. USA..

[30] Casano AM, Albert M, Peri F (2016). Developmental apoptosis mediates entry and positioning of microglia in the zebrafish brain. Cell Rep..

[31] Ma AC (2016). FusX: a rapid one-step transcription activator-like effector assembly system for genome science. Hum. Gene. Ther..

[32] Ma AC, Ward AC, Liang R, Leung AY (2007). The role of jak2a in zebrafish hematopoiesis. Blood.

[33] Ogryzko NV (2014). Zebrafish tissue injury causes upregulation of interleukin-1 and caspase-dependent amplification of the inflammatory response. Dis. Model. Mech..

[34] Bill BR, Petzold AM, Clark KJ, Schimmenti LA, Ekker SC (2009). A primer for morpholino use in zebrafish. Zebrafish.

[35] Taylor JC (2017). A novel zebrafish-based model of nociception. Physiol. Behav..

[36] Xu J, Wang T, Wu Y, Jin W, Wen Z (2016). Microglia colonization of developing zebrafish midbrain is promoted by apoptotic neuron and lysophosphatidylcholine. Dev. Cell..

[37] Wang X-W (2015). Plasma levels of high mobility group box 1 increase in patients with posttraumatic stress disorder after severe blunt chest trauma: a prospective cohort study. J. Surg. Res..

[38] Wingo AP (2018). Expression of the PPM1F gene is regulated by stress and associated with anxiety and depression. Biol. Psychiatry.

[39] Wu F (2016). Complement component C3a plays a critical role in endothelial activation and leukocyte recruitment into the brain. J. Neuroinflammation.

[40] Wang RP-H, Ho Y-S, Leung WK, Goto T, Chang RC-C (2019). Systemic inflammation linking chronic periodontitis to cognitive decline. Brain. Behav. Immun..

[41] Huang C (2019). Differential effects of propofol and dexmedetomidine on neuroinflammation induced by systemic endotoxin lipopolysaccharides in adult mice. Neurosci. Lett..

[42] Wohleb ES (2014). Re-establishment of anxiety in stress-sensitized mice is caused by monocyte trafficking from the spleen to the brain. Biol. Psychiatry.

[43] Wohleb ES, McKim DB, Sheridan JF, Godbout JP (2014). Monocyte trafficking to the brain with stress and inflammation: a novel axis of immune-to-brain communication that influences mood and behavior. Front. Neurosci..

[44] Wohleb ES, Powell ND, Godbout JP, Sheridan JF (2013). Stress-induced recruitment of bone marrow-derived monocytes to the brain promotes anxiety-like behavior. J. Neurosci..

[45] Cusick MF, Libbey JE, Patel DC, Doty DJ, Fujinami RS (2013). Infiltrating macrophages are key to the development of seizures following virus infection. J. Virol..

[46] Yong VW, Rivest S (2009). Taking advantage of the systemic immune system to cure brain diseases. Neuron.

[47] Bremner JD (2006). Traumatic stress: effects on the brain. Dialogues Clin. Neurosci..

[48] Rea V, Van Raay TJ (2020). Using zebrafish to model autism spectrum disorder: a comparison of ASD risk genes between zebrafish and their mammalian counterparts. Front. Mol. Neurosci..

[49] Paouri E, Tzara O, Kartalou GI, Zenelak S, Georgopoulos S (2017). Peripheral tumor necrosis factor-alpha (TNF-α) modulates amyloid pathology by regulating blood-derived immune cells and glial response in the brain of AD/TNF transgenic mice. J. Neurosci..

[50] Varvel NH (2016). Infiltrating monocytes promote brain inflammation and exacerbate neuronal damage after status epilepticus. Proc. Natl Acad. Sci. USA.

[51] Li S (2021). Microglial deletion and inhibition alleviate behavior of post-traumatic stress disorder in mice. J. Neuroinflammation.

[52] McMahon EJ, Suzuki K, Matsushima GK (2002). Peripheral macrophage recruitment in cuprizone-induced CNS demyelination despite an intact blood-brain barrier. J. Neuroimmunol..

[53] Ransohoff RM, Kivisäkk P, Kidd G (2003). Three or more routes for leukocyte migration into the central nervous system. Nat. Rev. Immunol..

[54] Jeong J-Y (2008). Functional and developmental analysis of the blood-brain barrier in zebrafish. Brain Res. Bull..

[55] Levy RM (2007). Systemic inflammation and remote organ injury following trauma require HMGB1. Am. J. Physiol. Regul. Integr. Comp. Physiol..

[56] Alam A (2020). Cellular infiltration in traumatic brain injury. J. Neuroinflammation.

[57] Huber-Lang M, Lambris JD, Ward PA (2018). Innate immune responses to trauma. Nat. Immunol..

