# Supplementary material for: Leukocyte invasion of the brain after peripheral trauma in zebrafish (Danio rerio)
Source: Exp Mol Med. 2022 Jul 13;54(7):973–87. doi: 10.1038/s12276-022-00801-4 (PMC9356012; doi:10.1038/s12276-022-00801-4)
Supplement: Supplementary file 1 — Supplementary figures [file 12276_2022_801_MOESM1_ESM.pdf]

## Supplementary information

Supplementary Table 1, Supplementary Fig. 1-6, and Supplementary Video 1-7.

## Supplementary Table

**Supplementary Table 1. List of primers used in the current study.**

| **Name** | **Sequence (5’-3’)** |
|---|---|
| ***Primers for Genotyping*** | |
| ZF_*il1b*_E4_GF | CGACATTTCACATCTGCACCG |
| ZF_*il1b*_E4_GR | GTACCTTGGATGACGTTCGC |
| ***Primers for qPCR*** | |
| ZF_*il1b*_QF | TGGACTTCGCAGCACAAAATG |
| ZF_*il1b*_QR | GTTCACTTCACGCTCTTGGATG |
| ZF_*tnfa*_QF | TTCACGCTCCATAAGACCCA |
| ZF_*tnfa*_QR | CCGTAGGATTCAGAAAAGCG |
| ZF_*il34*_QF | ATCGGACCATGCTTTGCCTG |
| ZF_*il34*_QR | CCCTACAACAGCAACACGGA |
| ZF_*il6*_QF | TGAAGACACTCAGAGACGAGCAGTT |
| ZF_*il6*_QR | AGGTTTGAGGAGAGGAGTGCTGAT |
| ZF_*il8*_QF | CCTGGCATTTCTGACCATCAT |
| ZF_*il8*_QR | GATCTCCTGTCCAGTTGTCAT |
| ZF_*il10*_QF | TCAGAGCAGGAGAGTCGAATGCA |
| ZF_*il10*_QR | CGATTGGGGTTGTGGAGTGCTT |
| ZF_*ccl2*_QF | GTCTGGTGCTCTTCGCTTTC |
| ZF_*ccl2*_QR | TGCAGAGAAGATGCGTCGTA |
| ZF_*lcp1*_QF | GAAGCTCTGATCGCTCTGCT |

| | |
|---|---|
| ZF_*lcp1*_QR | GTTGTTGATTTTGGGGCATC |
| ZF_*caspase3*_QF | TAGTGTGTGTGTTGCTCAGTC |
| ZF_*caspase3*_QR | CTCGACAAGCCTGAATAAAG |
| ZF_*b-actin*_QF | TTCCTTCCTGGGTATGGAATC |
| ZF_*b-actin*_QR | GCACTGTGTTGGCATACAGG |
| ***Primers for ISH Probes*** | |
| ZF_*il1b*_PF | TGCAATGCACGATTTGCGAT |
| ZF_*il1b*_PF | AGAATAAGCAGCACTTGGGGA |
| ZF_*il6*_PF | TCAGAGACGAGCAGTTTGAGAG |
| ZF_*il6*_PR | CCTCTTGGGGTCTTTCCCTC |

## Supplementary Figures

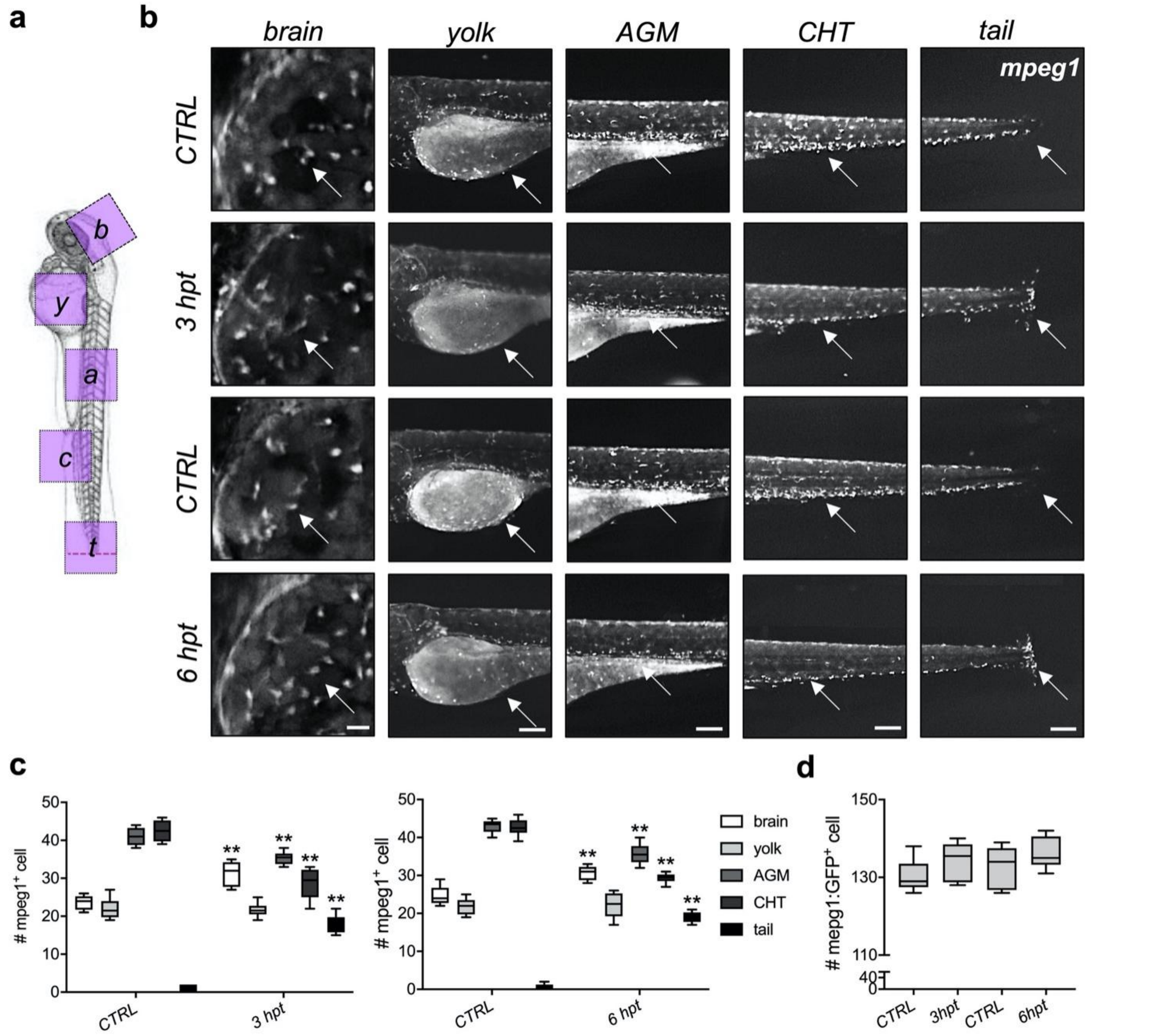


**Supplementary Fig. 1. Redistribution of macrophages in response to peripheral trauma.** (**a-c**) The number of *mpeg1*+ macrophages in various positions, including the brain (b), yolk (y), aorta-gonad-mesonephros (AGM, a), caudal hematopoietic tissue (CHT, c) and tail (t), and the total number of *mpeg1*+ macrophages (**d**) of zebrafish body at 3 hpt and 6 hpt. Independent t-test, **, $p < 0.01$ compared with CTRL. Scale bar, 40 μm (brain), 200 μm (yolk, AGM, CHT, and tail).

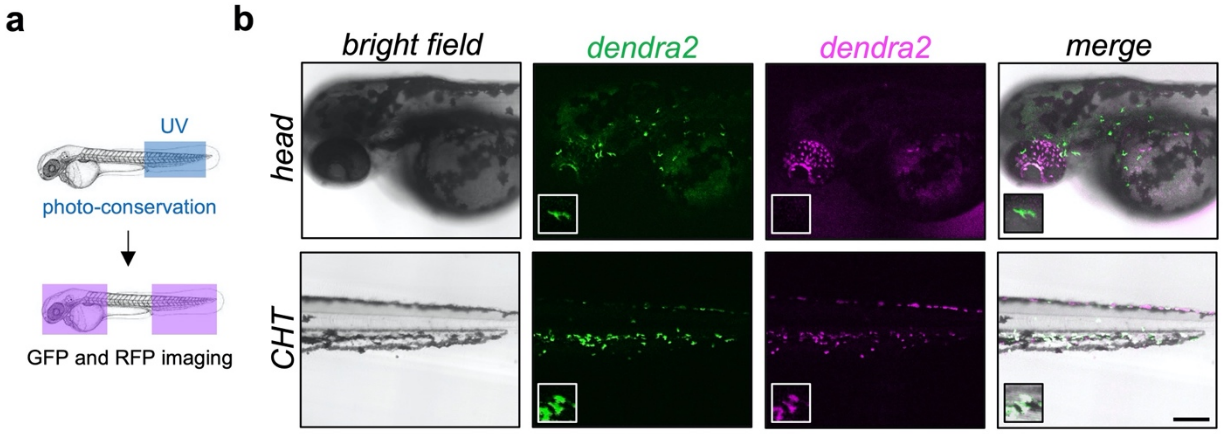


**Supplementary Fig. 2. Dendra2 photo-conservation and confirmation**. (**a**) Schematic diagram of the body regions for photo-conservation (tail/CHT) and confirmation (head and tail/CHT). (**b**) Photo-conserved magenta+ macrophages were detected in the tail/CHT region but not in the head region. Scale bar, 200 μm.

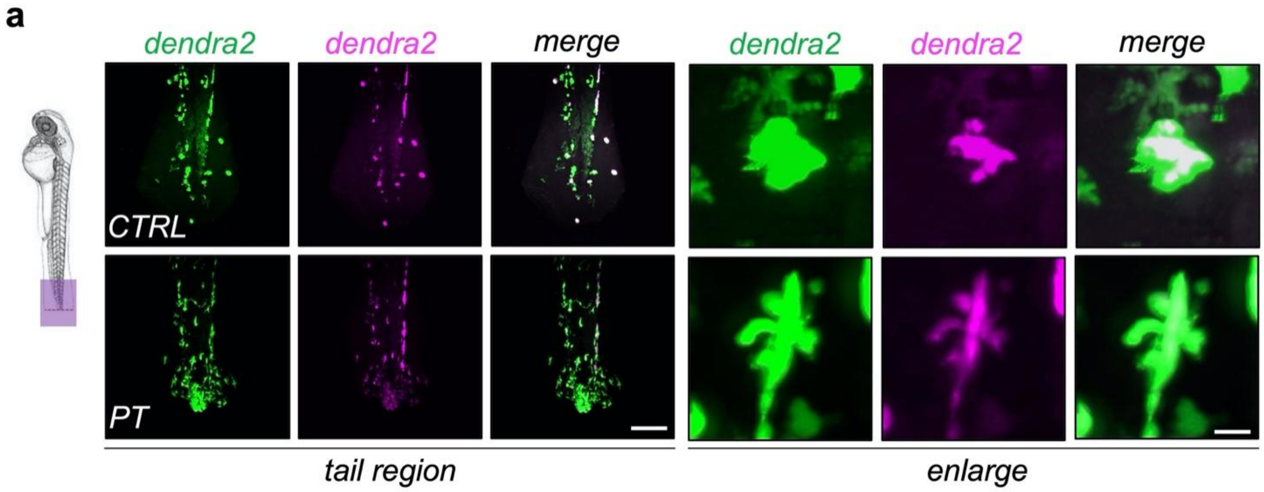


**Supplementary Fig. 3. Recruitment of photo-conserved macrophages in the traumatic site**. (**a**) Photo-conserved magenta+ macrophages were detected in the traumatic site of 4dpf zebrafish larvae. Scale bar, 200 μm, 20 μm (enlarge).

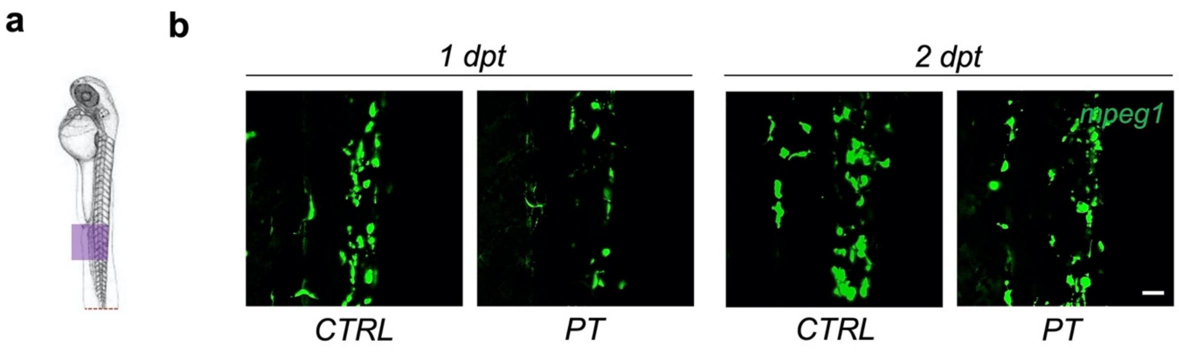


**Supplementary Fig. 4. Peripheral macrophages return to caudal hematopoietic tissue after peripheral trauma.** (**a-b**) Light-sheet imaging showed that *mpeg1*+ macrophages returned to caudal hematopoietic tissue at 2 days post-trauma. Scale bar, 100 µm.

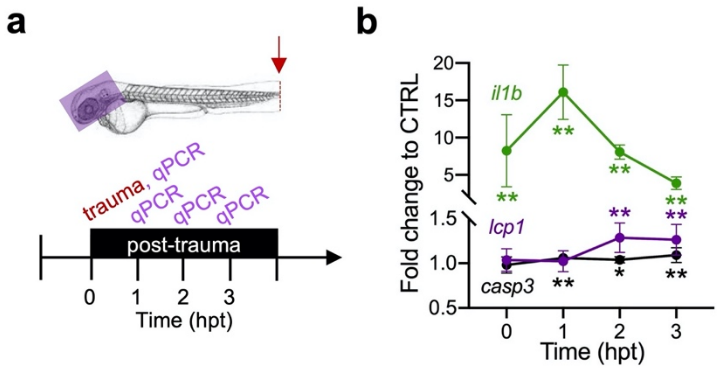


**Supplementary Fig. 5. Inflammatory, apoptotic, and leukocyte responses in the brain after peripheral trauma.** (**a**) Experimental setup. (**b**) qPCR results show that inflammatory cytokine (*il1b*), apoptosis (*caspase3*), and leukocyte invasion (*lcp1*) sequentially increased in the brain after peripheral trauma. One-way ANOVA, **, $p < 0.01$ compared with CTRL.

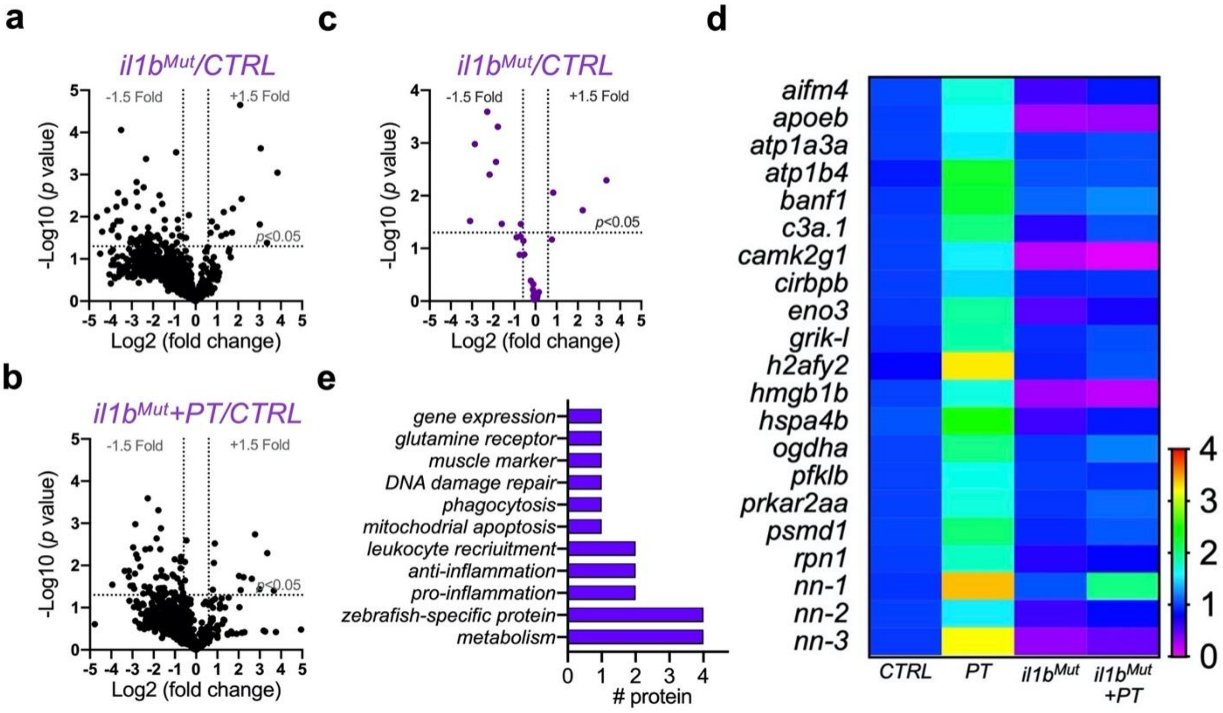


**Supplementary Fig. 6. Systemic inflammation-mediated brain proteomic responses to peripheral trauma.** (**a-b**) The volcano plot illustrated the proteins significantly ($p < 0.05$, independent t-test) changed (> 1.5 folds) in the brain at 1 dpt in *il1b*$^{Mut}$/CTRL and *il1b*$^{Mut}$+PT/CTRL. (**c**) The volcano plot illustrated the identified proteins significantly changed in the brain of *il1b*$^{Mut}$ zebrafish at 1 dpt. (**d**) Heatmap illustrated the fold change of identified proteins among CTRL, PT, *il1b*$^{Mut}$, and *il1b*$^{Mut}$+PT groups. (**e**) Classification of identified proteins into various molecular pathways or processes.

## Supplementary Videos

**Supplementary Video 1. Leukocyte activities in the brain of zebrafish larvae without peripheral trauma.** Red arrow, active leukocytes.

**Supplementary Video 2. Leukocyte activities in the brain of zebrafish larvae with peripheral trauma.** Red arrow, active leukocytes.

**Supplementary Video 3. Leukocyte activities in the brain of *il1b* mutant zebrafish larvae.** Red arrow, active leukocytes.

**Supplementary Video 4. Leukocyte activities in the brain of *il1b* mutant zebrafish larvae with peripheral trauma.** Red arrow, active leukocytes.

**Supplementary Video 5. Distribution pattern 1 of active leukocytes in the mid-hindbrain boundary of zebrafish larvae.** Red arrow, active leukocytes.

**Supplementary Video 6. Distribution pattern 2 of active leukocytes within the brain of zebrafish larvae.** Red arrow, active leukocytes.

**Supplementary Video 7. Distribution pattern 3 of active leukocytes migrating from the peripheral tissue into the brain.** Red arrow, active leukocytes.
